# Individually optimized estimation of energy expenditure in rescue workers using a tri-axial accelerometer and heart rate monitor

**DOI:** 10.3389/fphys.2024.1322881

**Published:** 2024-02-15

**Authors:** Hitomi Ogata, Yutaro Negishi, Nao Koizumi, Hisashi Nagayama, Miki Kaneko, Ken Kiyono, Naomi Omi

**Affiliations:** ^1^ Graduate School of Humanities and Social Sciences, Hiroshima University, Hiroshima, Japan; ^2^ Faculty of Health and Sport Sciences, University of Tsukuba, Tsukuba, Japan; ^3^ Graduate School of Engineering Science, Osaka University, Toyonaka, Japan

**Keywords:** rescue operations, metabolic equivalent of task, disaster simulation training, estimation equation, wearable

## Abstract

**Objectives:** This study aimed to provide an improved energy expenditure estimation for heavy-load physical labor using accelerometer data and heart rate (HR) measured by wearables and to support food preparation and supply management for disaster relief and rescue operations as an expedition team.

**Methods:** To achieve an individually optimized estimation for energy expenditure, a model equation parameter was determined based on the measurements of physical activity and HR during simulated rescue operations. The metabolic equivalent of task (MET), which was measured by using a tri-axial accelerometer and individual HR, was used, where two (minimum and maximum) or three (minimum, intermediate, and maximum) representative reference points were selected for each individual model fitting. In demonstrating the applicability of our approach in a realistic situation, accelerometer-based METs and HR of 30 males were measured using the tri-axial accelerometer and wearable HR during simulated rescue operations over 2 days.

**Results:** Data sets of 27 rescue operations (age:34.2 ± 7.5 years; body mass index (BMI):22.9 ± 1.5 kg/m^2^) were used for the energy expenditure estimation after excluding three rescue workers due to their activity type and insufficient HR measurement. Using the combined approach with a tri-axial accelerometer and HR, the total energy expenditure increased by 143% for two points and 133% for three points, compared with the estimated total energy expenditure using only the accelerometer-based method.

**Conclusion:** The use of wearables provided a reasonable estimation of energy expenditure for physical workers with heavy equipment. The application of our approach to disaster relief and rescue operations can provide important insights into nutrition and healthcare management.

## 1 Introduction

An accurate estimation of energy expenditure can provide important information and guidelines for nutrition and healthcare management of physical workers. In particular, disaster relief and rescue teams must prepare and transport their food supplies in advance. The higher the degree of energy deficiency, the lower the energy expenditure in activity, especially during high-intensity physical activity, and the more significant the decrease in energy expenditure (Martin et al., 2011). Based on the studies conducted by the U.S. military, the lack of energy promotes muscle damage and muscle soreness, decreases the performance of physical activities (Margolis et al., 2014), causes weight loss and a greater percentage of muscle mass loss than fat mass (Berryman et al., 2017), decreased immune function (T lymphocyte response) (Kramer et al., 1997), and decreased cardiac function (altered left ventricular diastolic function) (Planer et al., 2012). Therefore, the total amount of food supplied must be sufficient to meet the nutritional needs of the body and to maintain the energy required for high-intensity physical activity. However, energy expenditure estimation methods for such workers have not been well established, and the energy expenditure during such operations remains unclear.

With recent advancements in sensor technologies, portable devices are becoming smaller, capable of longer data collection (because of their storage and battery lives), multidimensional, and more sensitive (Chen et al., 2012). Many movement sensors can be used to measure human physical activities, including electromechanical switches (for heel strike detection), mercury switches, pedometers, inclinometers, gyroscopes, goniometers (for angles or postures), accelerometers, and even global positioning systems (GPS) (Chen et al., 2012). Among these devices, accelerometers are currently the most widely used sensors for human physical activity monitoring in clinical and free-living settings (Chen et al., 2012) because of their small size, noninvasiveness, and relatively low cost (Plasqui and Westerterp, 2007). Moreover, accelerometers can easily estimate the amount of energy expenditure during high-intensity physical activities, except for heavy equipment and static load activities (Bouten et al., 1997). However, discriminating movements during daily low-intensity physical activities and estimating the appropriate energy expenditure remain a challenge (Ndahimana and Kim, 2017). Some tri-axial accelerometers have shown potential application in estimating the amount of energy expenditure during low-intensity physical activities, such as sitting, standing, housework, and walking, which are important for estimating total energy expenditure (Midorikawa et al., 2007; Yamada et al., 2009; Ohkawara et al., 2011). When measuring the total energy expenditure in field validation studies (double-labeled water (DLW), which is the gold standard for measuring total energy expenditure in the field), high correlations with the total energy expenditure estimate were found in most activity monitors (Van Remoortel et al., 2012). However, these correlations are to a large extent driven by subject characteristics, body weight, age, and height, which are important predictors of total energy expenditure (Plasqui et al., 2005). Based on previous reports, only 19% of the total energy expenditure is accounted for by physical activity in healthy subjects (Plasqui et al., 2005) and in patients with coronary heart disease (Ades et al., 2005) in a field setting. Previous research demonstrated that wearable devices, such as movement sensors, do not accurately represent energy expenditure measured by the DLW (Murakami et al., 2019; Willis et al., 2022). Several studies have also compared the DLW method to the total energy expenditure estimated by using accelerometers. The total energy expenditure using an accelerometer in patients with reduced pulmonary function, particularly chronic obstructive pulmonary disease, is underestimated by 11% compared with the DLW (Sato et al., 2021). Even the total energy expenditure of people who are not engaged in heavy physical activity is underestimated compared with that of accelerometer. A wrist-mounted motion sensor accounts for 78% of the variation of total energy expenditure using the DLW during the free-living period and 62% during the training period (Kinnunen et al., 2019). The total energy expenditure using an accelerometer in firefighters under normal working conditions is underestimated by 33% compared with the DLW (Touno et al., 2003). In the literature, the total energy expenditure by the accelerometer is underestimated compared with the DLW (Touno et al., 2003; Kinnunen et al., 2019; Murakami et al., 2019; Sato et al., 2021; Willis et al., 2022) despite the difference between wrist-mounted and waist/chest-worn accelerometers (Kinnunen et al., 2019). Therefore, the energy expenditure estimation for heavy equipment and static load activities has an underestimation bias, although accelerometer-based methods are convenient and applicable to real-world situations (Touno et al., 2003).

Various wearable devices to monitor heart rate (HR) have been developed, such as wristwatches, ear clips, and undershirts, and some devices can measure HR accurately. These devices can record HR noninvasively, with minimal technical effort and without the constraints of laboratory conditions (Karmen et al., 2019; Isakadze and Martin, 2020). The HR increases almost proportionally to exercise intensity and intra-individual oxygen uptake (% ${\dot{V}O}_{2}\max$ ), and its dynamics can influence a number of different factors, such as body temperature, food intake, body posture, and individual cardio-respiratory fitness level (Hill and Trowbridge, 1998). However, the use of HR wearable devices, most of which are wrist-worn devices, may underestimate energy expenditure and provide inaccurate measures of energy expenditure compared with reference standard criterion measures, including direct calorimetry and indirect calorimetry (Fuller et al., 2020; Chevance et al., 2022). Summarizing the total energy expenditure by most of the aforementioned devices, such as accelerometer, HR, GPS, and combined motion sensors, provides a more accurate estimation of energy expenditure at light-to-moderate intensities; by contrast, underestimation increases at very light and higher intensity activities (Aparicio-Ugarriza et al., 2015).

A previous review reported that adding indicators such as HR and heat flux values to acceleration values as a method of estimating daily energy expenditure and physical activity has not significantly improved the system (Van Remoortel et al., 2012). Nevertheless, focusing on the HR that can be measured with a wearable device can address the underestimation of energy expenditure by using only a tri-axial accelerometer because HR can relate intensity to energy expenditure. This study aimed to provide an energy expenditure estimation method using wearables to support food preparation and supply management, particularly for expedition rescue team workers with heavy equipment and static loads. By combining the characteristics of tri-axial accelerometers and HR monitor information, we evaluated the relationship between accelerometer-based physical activity and HR and provided an individually optimized energy expenditure estimation equation (model). Providing an equation/model to predict energy expenditure would promote the estimation of energy expenditure in the field, which is not measurable in the laboratory. As an application of this approach, the energy expenditure of rescue operations was estimated while mimicking large-scale disasters.

## 2 Materials and methods

### 2.1 Subjects

This study included 30 males (age: 34.2 ± 7.3 years; body mass index (BMI): 23.1 ± 1.7 kg/m^2^) who participated in a 2-day disaster simulation training (i.e., rescue operations were unaware of the training protocol). Note that some of the firefighters of our previously reported paper were included in the present study (Koizumi et al., 2021). This study was approved by the local ethics committee of the University of Tsukuba (approval number: tai-28-66), conducted in accordance with the principles set forth in the Declaration of Helsinki, and the subjects were informed of and agreed to the details of this study. In addition, detailed explanations were given to each training director and participating organization-affiliated institution regarding the purpose and content of the experiment, and the experiment was started only after obtaining agreement.

### 2.2 Simulated rescue operations

Rescue operation training was conducted from November 30 to 1 December 2018, in Kanagawa Prefecture, Japan. The mean temperature of the 2-day training was 12.8°C (8.5°C–18.3°C), with humidity of 55.5%. The main activities were as follows: rescue activity training in a skyscraper, in a gas leak accident, in a collapsed building, at a sediment-related disaster site, in the event of many injured people on the subway, from vehicles, and in a tunnel collapse accident; fire-extinguishing activity training in an industrial complex fire.

### 2.3 Measurements

#### 2.3.1 Accelerometer

**FIGURE 1:**
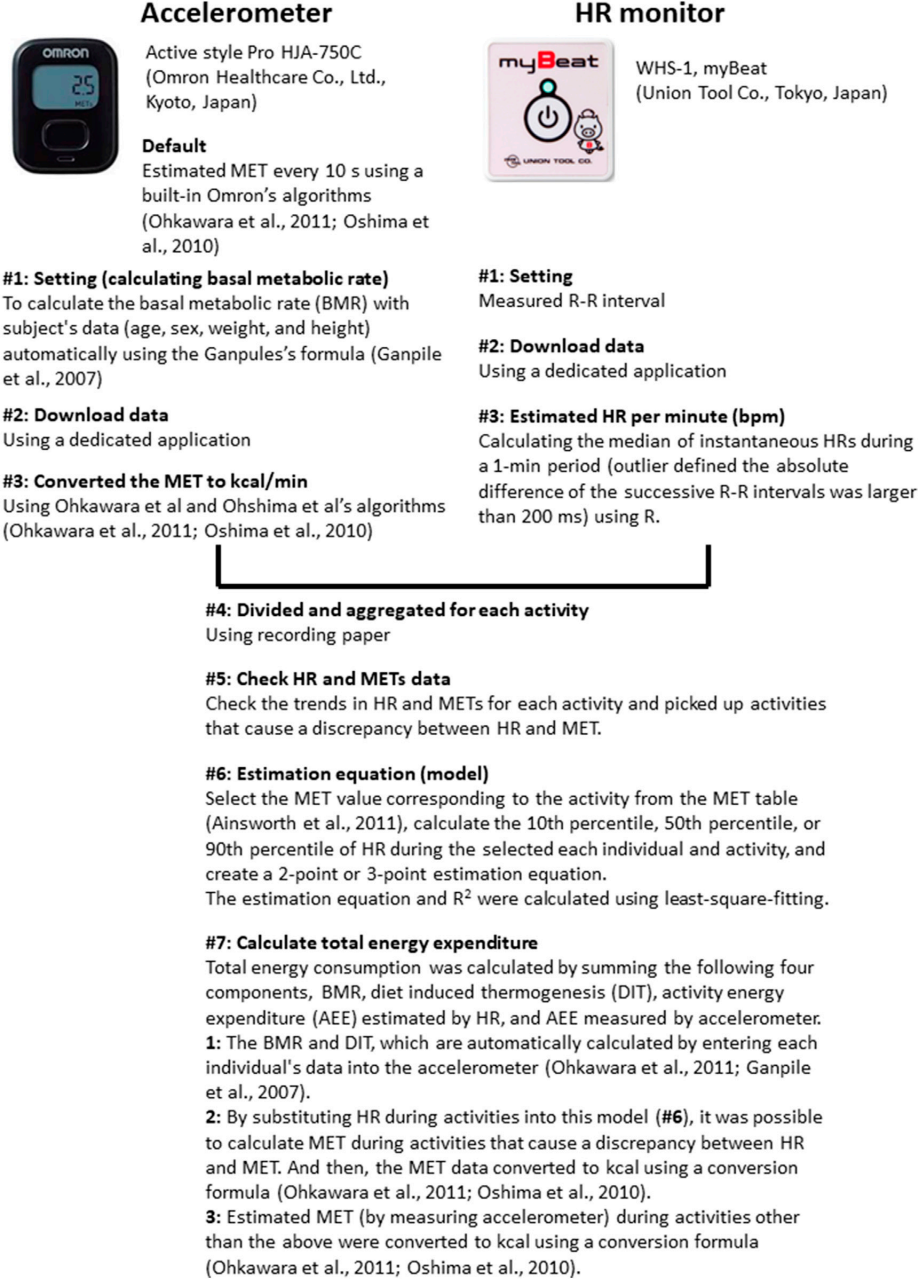
Flow chart of calculating energy expenditure.

Figure 1 shows flow chart of calculating energy expenditure. A tri-axial accelerometer (Active style Pro HJA-750C, 23 g, 40 mm × 52 mm × 12 mm; Omron Healthcare Co., Ltd., Kyoto, Japan) (Ohkawara et al., 2011; Nagayoshi et al., 2019; Nishida et al., 2020) was inserted into the chest pocket, located near the waist area (approximately 10 cm above the belt). The accelerometer was covered with a vinyl material and fixed in the chest pocket with adhesive tape to avoid the influence of liquid matter (e.g., water) in fire-extinguishing activities. The basic metabolic rate was calculated automatically when weight, height, age, and sex were entered, which was calculate using Ganpule’s formula (Ganpile et al., 2007). The accelerometer was programmed to save the metabolic equivalent of task (MET) once every 10 s using a built-in Omron’s algorithm (Oshima et al., 2010; Ohkawara et al., 2011). MET is defined as the ratio of the metabolic rate during an activity to the metabolic rate at rest (Jetté et al., 1990). Next, each transformed data point (METs) was converted into units of activity energy expenditure in kcal/min (Oshima et al., 2010; Ohkawara et al., 2011) to understand the amount of energy intake, and the data were divided and aggregated for each activity (see Section 2.3.2 Recording paper).

#### 2.3.2 Recording paper

A recording paper was distributed, and rescue squads were asked to describe the activities that they performed during the disaster simulation training. This recording paper was used to classify the types and times of activities.

#### 2.3.3 HR monitor

**FIGURE 2:**
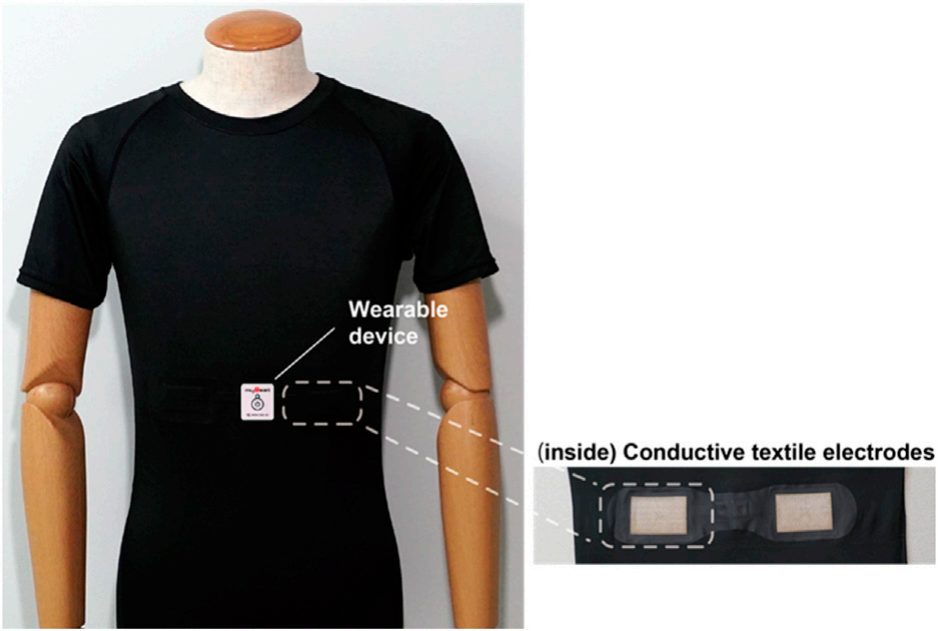
T-shirt attached with conductive fiber electrodes (backside). Stretchy shirts with electrodes were placed in the psoas. The size of the T-shirt was selected on the basis of the physique of the rescue operations.

R–R intervals were measured by electrocardiogram signals using wearable HR sensors (WHS-1, myBeat, 14 g, 39 mm × 37 mm × 9 mm; Union Tool Co., Tokyo, Japan) (Arikawa et al., 2020). The HR sensor was placed on a shirt with attached conductive fiber electrodes (Kurabo Industries Ltd., Osaka, Japan, Figure 2). The HR per minute [beats per minute (bpm)] was estimated using the median of instantaneous HRs during a 1-min period to reduce the effect of the outliers presented in R–R interval data. If the absolute difference of the successive R–R intervals was larger than 200 ms, then the detected R–R interval was classified as an outlier using R version 3.6.0 (R Foundation for Statistical Computing, Vienna, Austria; http://www.R-project.org/). The percentage of the outlier R–R interval for each individual was also calculated.

#### 2.3.4 Estimation equation (model)

A percent of HR reserve (%HRR) provides a good approximation (almost linear relation) of ${\dot{V}O}_{2}$ reserve ( $\%{\dot{V}O}_{2}$ ) was shown in the previous studies (Swain et al., 1998; Strath et al., 2000), and an HR-based equation can provide a reliable estimation of the METs (Swain et al., 1998; Strath et al., 2000). %HRR is defined as follows:

$$\%\text{HRR}=\frac{{\text{HR}}_{\text{activity}}-{\text{HR}}_{\text{rest}}}{{\text{HR}}_{\max}-{\text{HR}}_{\text{rest}}}\times 100$$

where ${\text{HR}}_{\text{activity}}$ is the recorded HR during the activity; ${\text{HR}}_{\text{rest}}$ is the HR while sitting at rest, and HRmax is the maximum HR as estimated by the Karvonen equation, (220 bpm—age) bpm. $\%{\dot{V}O}_{2}$ reserve is defined as follows:

$$\%{\dot{V}O}_{2}R=\frac{{\dot{V}O}_{2\text{act}}-{\dot{V}O}_{2\text{rest}}}{{\dot{V}O}_{2\max}-{\dot{V}O}_{2\text{rest}}}\times 100,$$

where ${\dot{V}O}_{2\text{activity}}$ is the recorded $\dot{V}O_{2}$ during the activity; ${\dot{V}O}_{2\text{rest}}$ is the $\dot{V}O_{2}$ while sitting at rest, and ${\dot{V}O}_{2\max}$ is the maximum $\dot{V}O_{2}$ as estimated by the equation proposed by Jackson et al. (1990). By assuming %HRR≈ $\%{\dot{V}O}_{2}R$ and dividing the numerator and denominator of the $\%{\dot{V}O}_{2}R$ by the ${\dot{V}O}_{2\text{rest}}$ , we obtain the following equation:

$$\frac{{\text{HR}}_{\text{act}}-{\text{HR}}_{\text{rest}}}{{\text{HR}}_{\max}-{\text{HR}}_{\text{rest}}}=\frac{\frac{{\dot{V}O}_{2\text{act}}}{{\dot{V}O}_{2\text{rest}}}-1}{\frac{{\dot{V}O}_{2\max}}{{\dot{V}O}_{2\text{rest}}}-1}=\frac{{\text{METS}}_{\text{act}}-1}{{\text{METS}}_{\max}-1}$$

Based on the abovementioned relation, we obtain the following equation for MET estimation (Strath et al., 2000):

$${\text{METS}}_{\text{act}}=\frac{{\text{METS}}_{\max}-1}{{\text{HR}}_{\max}-{\text{HR}}_{\text{rest}}}\left({\text{HR}}_{\text{act}}-{\text{HR}}_{\text{rest}}\right)+1$$

Therefore, if we use two reference conditions with different METs, ${\text{METS}}_{1}$ and ${\text{METS}}_{2}$ , instead of resting and the maximum $\dot{V}O_{2}$ condition, then we can obtain a more general equation for MET estimation.

$${\text{METS}}_{\text{act}}=\frac{{\text{MET}}_{2}-{\text{MET}}_{1}}{{\text{HR}}_{2}-{\text{HR}}_{1}}\left({\text{HR}}_{\text{act}}-{\text{HR}}_{1}\right)+{\text{METS}}_{1},\left(*\right)$$

where ${\text{HR}}_{1}$ and ${\text{HR}}_{2}$ are the HR recorded during the activity with ${\text{METS}}_{1}$ and ${\text{METS}}_{2}$ , respectively.

**FIGURE 3:**
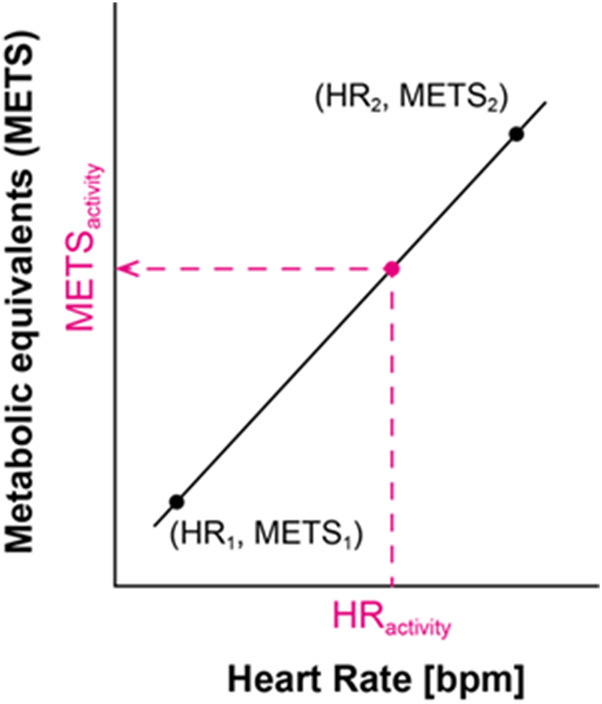
Estimation equation between HR and METs.

We used two (minimum and maximum) or three (minimum, intermediate, and maximum) representative reference points recorded in each individual (Figure 3) to obtain the parameters, ( ${\text{HR}}_{1},{\text{MET}}_{1}$ ), ( ${\text{HR}}_{2},{\text{MET}}_{2}$ ), in the MET estimation equation [Eq (*)] optimized to each individual. The lowest HR, ${\text{HR}}_{1},$ corresponding ${\text{MET}}_{1}$ was calculated as follows: 1) the 10th percentile of HR during napping was calculated as ${\text{HR}}_{1}$ , and 2) we defined the ${\text{MET}}_{1}$ as 0.95 MET based on the MET table (Garby et al., 1987). We selected the 10th percentile of HR instead of the observed lowest value to attenuate the effect of outliers of the HR measurement. The intermediate HR was calculated as follows: First, the 50th percentile of HR during withdrawal was calculated as the intermediate HR because withdrawal was performed by all groups and was considered to be less affected by tension as it was performed after all activities were completed, and then we defined the intermediate HR as 3.0 MET based on the MET table (Ainsworth et al., 2011). Moreover, the highest HR corresponding to the highest MET during all activities was calculated as follows: First, the 90th percentile of HR was calculated for each rescue operation activity. Although the highest HR would be induced by the activity with the highest MET, we selected the 90th percentile of HR instead of the observed highest value to attenuate the effect of outliers of the HR measurement. Second, activity’s METs were defined on the basis of the MET table (Table 1) (Ainsworth et al., 2011), and then the activity with the highest HR was selected. Finally, the parameters, ( ${\text{HR}}_{2},{\text{MET}}_{2}$ ), were estimated for all participants using a least-square-fitting on the abovementioned values.

**TABLE 1:** Activities corresponding to the MET table.

Code	Major heading	Activity^a^ (description)	METs
05147	home activities	withdrawal (implied walking, putting away household items, moderate effort)	3.0
11240	occupation	rescue narrow space, rescue activity, rescue search (fire fighter, general)	8.0
11244	occupation	rescue traffic, rescue transport (fire fighter, rescue victim, automobile accident, using pike pole)	6.8
11245	occupation	fire training (fire fighter, raising and climbing ladder with full gear, simulated fire suppression)	8.0
11246	occupation	gas rescue, rescue gas search, rescue gas transport (fire fighter, hauling hoses on ground, carrying/hoisting equipment, breaking down walls, wearing full gear)	9.0
11550	occupation	rescue sediment, rescue tunnel (shoveling, more than 7.3 kg/min, deep digging, vigorous effort)	8.8
17029	walking	rescue activity training in a skyscraper (carrying 22.7–33.6 kg load, upstairs)	10.0

^a^ Activity represents the rescue operations conducted in this study. The code, major heading, description, and METs, are provided in the MET, table (Ainsworth et al., 2011).

These two rescue squads were excluded from analysis because one rescue squad was only able to measure their HR for 15% during their nap time, and the activity of another rescue squad did not include withdrawal.

#### 2.3.5 Statistical analysis

Data are presented as mean values and standard deviations.

## 3 Results

### 3.1 Typical example of measurements

**FIGURE 4:**
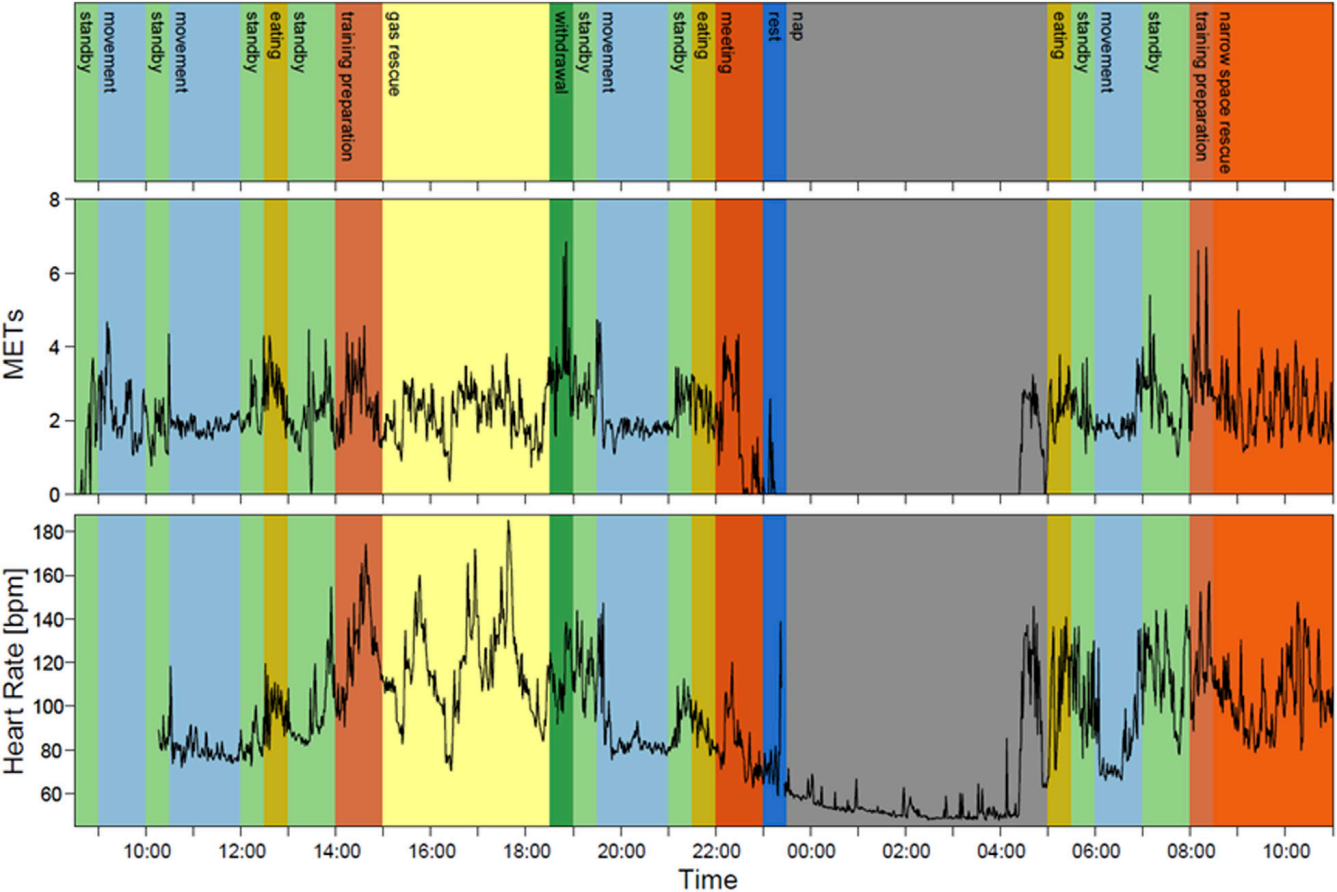
Typical result of METs and HR for one rescue worker during the 2-day disaster simulation training. The rescue worker engaged in rescue activity training in a gas leak accident (yellow) and excavation rescue in a narrow space (light orange). Colors were categorized for each activity: gray, nap; orange, meeting; light blue, movement; blue, rest; ocher, eating; light green, standby; green, withdrawal; yellow, gas leak accident; light orange, rescue narrow space; and brown, training preparation.

We measured the physical activity and HR from 8:30 to 11:00 the next day. Figure 4 represents a typical example of METs and HR during simulated rescue operations (28 years, 173 cm, 72 kg). The rescue worker engaged in rescue activity training in a gas leak accident (yellow) for 210 min, excavation rescue in narrow space (light orange) for 150 min, and nap (gray) for 330 min. The basic metabolic rate of the rescue worker, which is automatically calculated, was 1,600 kcal, and the total energy expenditure estimated by using the tri-axial accelerometer was 3,723 kcal (from 11:00 to 11:00 the next day). This example underestimated METs in the yellow and light-orange areas because the activities did not show high METs despite an increase in HR. Therefore, METs are more stable than HR during low-intensity activities, particularly during napping.

### 3.2 Calculation of HR for each activity

One rescue worker who conducted excavation rescue search and tunnel accident rescue activities could measure only the first 4 h (deficiency rate was 83.1%). Thus, the rescue worker was excluded from analysis. The percentage of missing HR data for each individual was 4.7% ± 4.4% (range: 0%–15.3%) for 27 rescue squads (age: 34.2 ± 7.5 years; BMI: 22.9 ± 1.5 kg/m^2^). The HR was computed as the 10th percentile for naps, 50th percentile for withdrawal, and 90th percentile for other activities. The number of subjects in each activity is summarized in Table 2. Nap had the lowest HR, and rescue activity training in a skyscraper had the highest HR.

**TABLE 2:** Heart rate during each activity.

Activity (METs)^a^	Heart rate (bpm)^b^	*n*
Nap (0.95 METs)	56.2 ± 13.5	27
Withdrawal (3.0 METs)	104.8 ± 17.2	27
Rescue training from vehicles (6.8 METs)	121.9 ± 24.1	5
Search activity (6.8 METs)	125.4 ± 20.7	10
Rescuer transport (6.8 METs)	132.9 ± 16.9	5
Rescue training in a collapsed building (8.0 METs)	116.7 ± 20.3	15
General firefighting (8.0 METs)	132.5 ± 14.6	5
Excavation rescue search activity (8.8 METs)	128.2 ± 13.8	9
Tunnel accident rescue activity (8.8 METs)	131.2 ± 15.7	4
Rescue activity training in a gas leak accident (9.0 METs)	131.2 ± 21.2	15
General rescue activity (9.0 METs)	135.1 ± 17.8	10
Rescue activity training in a skyscraper (10.0 METs)	154.9 ± 23.9	20

The results are presented as mean values ±SD.

^a^ The table shows the activities extracted to create the equations for the relationship between HR, and METs; the figure is based on the MET, table (Garby et al., 1987; Ainsworth et al., 2011).

^b^ HR, which was computed as the 10th percentile for nap, 50th percentile for withdrawal, and 90th percentile for other activities, is also shown. The number of rescue operations engaged in the activity is depicted as *n.*

### 3.3 Basic metabolic rate and estimated total energy expenditure by the tri-axial accelerometer

The tri-axial accelerometer automatically calculated the basic metabolic rate, with an average of 1,545 ± 92 kcal, and the estimated total energy expenditure, with an average of 3,414 ± 229 kcal for 27 rescue squads (from 11:00 to 11:00 the next day).

### 3.4 Estimated total energy expenditure by combining the tri-accelerometer and HR

**FIGURE 5:**
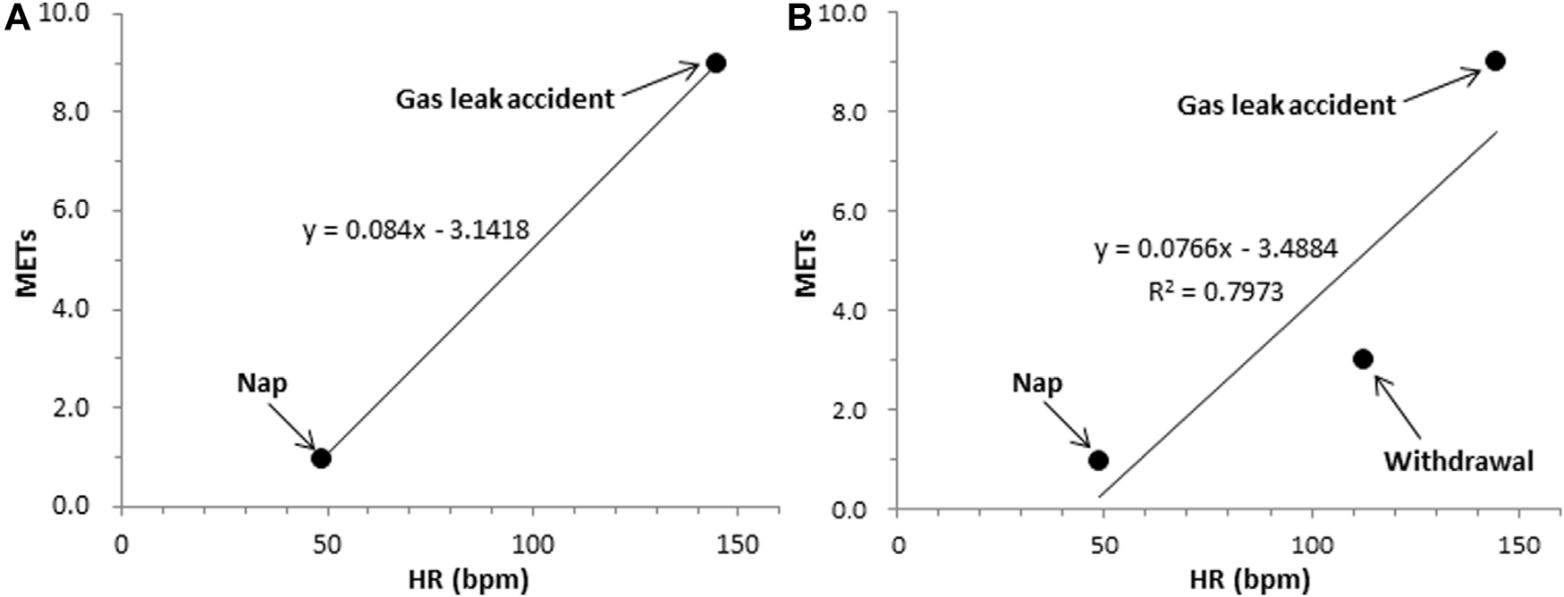
Estimation equation between HR and METs for a typical example of one rescue worker (Figure 4). **(A)** two points; **(B)** three points.

Figure 4 shows the estimation equation using two points (Figure 5A) created using the 10th percentile of HR during nap (0.95 METs) as the lowest HR and the 90th percentile of HR during rescue activity training in a gas leak accident (9.0 METs). Using three points (Figure 5B), the estimation equation was created using the abovementioned two points plus the intermediate 50th percentile of the HR during withdrawal (3.0 METs). The results showed that HR was higher during rescue training in a gas leak accident (9.0 METs) than during the excavation rescue in a narrow space (6.8 METs). Therefore, we developed an equation to estimate the highest HR for the latter (Figure 4). The energy expenditure calculated using the estimation equation replaced specific rescue activities, such as rescue activity training in a gas leak accident (yellow) and excavation rescue in a narrow space (light orange). The estimated total energy expenditure was 5,456 kcal using the two-point equation and 4,945 kcal using the three-point equation. The difference between the estimated total energy expenditure by the tri-axial accelerometer and that by combining the tri-axial accelerometer and HR was 1,733 kcal using the two-point equation and 1,222 kcal using the three-point equation.

The average value of *R* ^2^ in the estimation equation was 0.895 (95% confidence interval: 0.858–0.932) using the three-point equation (minimum, intermediate, and maximum). The average corrected total energy expenditure obtained by the tri-axial accelerometer and HR was 4,871 ± 486 kcal and 4,555 ± 391 kcal, respectively. The average difference between the estimated total energy expenditure by the tri-axial accelerometer and that by combining the tri-axial accelerometer and HR was 1,457 ± 486 kcal and 1,140 ± 410 kcal, respectively (accounting for an average increase of 143% and 133%, respectively).

## 4 Discussion

In this study, we developed a simple energy expenditure estimation method using wearables (tri-axial accelerometer and HR) for heavy load physical laborers to achieve a reliable estimation of energy expenditure for disaster relief and rescue workers as an expedition team. The relationship between the MET and individual HR in humans was also explored. If the minimum HR is known, then the maximum HR can be largely predicted; therefore, a model with some uniformity can be created without laboratory assessment. The strength of this study depends on the measurement of tri-axial acceleration, which can isolate each activity, capture a wide range of movement, as well as HR for rescue workers who experience various difficulties at the forefront, and estimate the total energy expenditure for each individual using a novel method in the field.

The MET concept, which expresses the intensity of physical activity by the time it corresponds to the resting metabolic rate, represents a simple, practical, and easily understood procedure to express the energy cost of physical activities as a multiple of the resting metabolic rate (Jetté et al., 1990); one MET represents the state of sitting and resting. In the present study, an accelerometer was used to store MET because energy expenditure can be calculated by multiplying the MET by a coefficient and individual body weight. The HR is high under the influence of mental and physical fatigue (Tanaka et al., 2011), stress, tension, and excitement (Cannon, 1929). Many researchers will conduct standardized laboratory assessments of resting and maximal HR before field testing using the submaximal test and/or Yo–Yo intermittent endurance test to determine maximal HR in the field (Sell and Ledesma, 2016). This approach is advantageous because the HR data in the field can be normalized to each individual, and other researchers can easily replicate those standardized tests. However, if only the minimum HR is known in humans, then the maximum HR can be largely predicted, that is, %HRR. Consequently, an estimation equation model with some uniformity can be created. Previous research demonstrated that estimating the total energy expenditure by using only an accelerometer is underestimated compared with the DLW (Touno et al., 2003; Kinnunen et al., 2019; Murakami et al., 2019; Sato et al., 2021; Willis et al., 2022). Therefore, given the nature of accelerometers, accurately measuring the total energy expenditure during heavy equipment or static load activities is difficult (Touno et al., 2003). In the present study, we combined METs and HR to improve underestimation.

METs differ depending on posture; therefore, we hypothesized that the METs during sitting and resting are different. Thus, 0.95 MET was derived from the original paper (Garby et al., 1987) of METs for a nap. We defined the minimum HR as the 10th percentile of nap time because the participating rescue squads operating in a blind training environment where they did not know what to do next slept very differently than usual and slept on their cots. In this study, the average HR during nap was 68.3 ± 15.3 bpm/min, which may also be affected by the abovementioned factors. The maximum HR was set at the 90th percentile of each rescue operation’s activity because the same HR did not last forever, although the same activity was continued. Activity and activity time were classified on the basis of self-reported recording forms; however, the actual time spent waiting was also included, which may have reduced the HR during the activity. Although the training was conducted at relatively low temperatures in winter, the HR, which was set at the 90th percentile for training in winter conditions, may need to be higher because the load on the body is expected to be higher in summer than in winter. As rescue operations include various activities, not all activities can be applied to appropriate METs. However, a positive correlation was observed between METs and HR, indicating that the wearable device could accurately measure HR without burdening the subject. The HR values during multiple activities were consistent with those reported in previous studies (Rodahl, 1989; Parker et al., 2017). Average HRs during all tasks ranged from 110 to 130 bpm (Rodahl, 1989) and from 145 to 109 bpm for steep and flatlands, respectively (Parker et al., 2017). We defined withdrawal as an intermediate HR as it was an activity carried by all groups. In most rescue squad estimation equations, the intermediate point shifts downward. In this study, the METs may not be appropriate because withdrawal includes activities such as carrying heavy loads, moving, and feeling fatigue. Based on the error propagation law, the error in the estimated ${\text{METS}}_{\text{act}}$ depends on the accuracy of the measurements in METs and HR at the assumed maximum intensity. Improving the accuracy of those measurements with the aid of laboratory measurements is a future challenge.

A previous systematic review reported that tri-axial and multisensory devices tend to be more valid monitors compared with the DLW while adding indicators such as HR and heat flux values to acceleration values to estimate daily energy expenditure, and physical activity has only slightly improved the system (Van Remoortel et al., 2012). In addition, the combination of motion sensor and HR is valid for estimating free-living energy expenditure compared with the DLW, but it is less accurate for an individual assessment (Sliva et al., 2015). Kortelainen et al. (2021) proposed accurate energy expenditure predictions based on a few calibration measurements using a nonlinear (logistic) mixed model for energy expenditure and HR. They found that the logistic mixed model performed better than the linear mixed model when predicting energy expenditure at population level and with calibration. In this study, using the linear model, the result of the estimated equation with three points was 94% (−316 kcal) compared with the results of the two points. Therefore, no moderate-intensity activity that did not affect HR. In addition, using the tri-axial accelerometer method, the estimation equation with two points of total energy expenditure was underestimated by 43% compared with the combined accelerometer and HR. In the present study, we able to improve the underestimation of total energy expenditure, but we could not examine whether it was an overestimation or an accurate assessment. The novel estimation equation of the two points between the METs and HR is simple, and it has high validity as an estimation equation for energy expenditure in the field. Considering its application in the field, the two minimum and maximum estimation equations used in this study are sufficient. In estimating the energy expenditure per activity for a short period, rather than the average energy expenditure for 1 day or several days, combining tri-axial accelerometers and HR was easy and effective. Our research objective was to estimate energy expenditure in a real-world environment. Since our approach was a real-world data-driven study, rather than a traditional experimental design-driven study, there were many limitations on the measurement. Under such real-world, non-uniform, and *a priori* unforeseeable work environments, we believe that our method gave improved estimation results compared to the results of previous studies that estimated using only acceleration. Also, the results we refer to are the widely-accepted reference values, METs, estimated based on experimental studies in previous studies. Nevertheless, indeed, the values estimated based on experimental studies do not take into account factors such as individual differences, mental load, different types of exercise load, the type of heavy equipment, effects of circadian rhythm, effects of diet, and effects of sleep. It is difficult to conduct an experiment that takes all these factors into account. We also believe that in real-world, non-uniform, and *a priori* unforeseeable work environments, accurate estimation of individual tasks is difficult, whether using the DLW or other methods. We are sure that the accuracy can be improved by combining it with laboratory studies in the future. Beckner et al. (2023) reported sustained operations for the military personnel are often conducted in a state of negative energy balance and are associated with degraded cognitive performance and mood. In order to prevent such condition, the amount of energy ingested should also be considered in the future. The accuracy of the estimation equation can be further improved by accurately extracting the reference activities of METs. Moreover, estimating energy expenditure for a wide range of age groups, sex, and people with different body sizes, such as obese and overweight, may require an estimation equation with three or more points, instead of just two, in the future.

This study had four limitations. First, we focused only on HR because the measurements were conducted in winter. However, the temperature should also be considered because the HR can quickly increase depending on environmental conditions, particularly during summer. In addition, an equation that considers the circadian rhythm of the heartbeat should be developed. Second, the activities were calculated by applying them to the MET table; however, applying them to special rescue operation activities was difficult because appropriate METs could not be found. Therefore, the validity of METs remains to be examined using the Douglas bag and other methods. Third, we did not consider the diet induced thermogenesis, because it was automatically calculated by entering each individual’s data into the accelerometer. Finally, the present study was conducted in the field, and no controls were used to verify the energy expenditure calculations. In the future, the energy expenditure remained to be examined using the Douglas bag and DLW.

In this study, a method to improve energy expenditure estimation for heavy-load physical labor, such as disaster relief and rescue operations, using a tri-axial accelerometer and HR monitor was proposed. Energy expenditure, which was underestimated by accelerometer-based energy expenditure methods, could be compensated by creating an individually optimized estimation equation between METs and individual HR. Furthermore, more detailed measurements were necessary for a large number of rescue operations in a wide range of ranks and firefighter activities in the future.

## Data Availability

The datasets presented in this study can be found in online repositories. The names of the repository/repositories and accession number(s) can be found below: All data files are available from the figshare database (https://figshare.com/articles/dataset/Estimation_equation_model_/21579168).
